# Effects of Tannic Acid Supplementation of a High-Carbohydrate Diet on the Growth, Serum Biochemical Parameters, Antioxidant Capacity, Digestive Enzyme Activity, and Liver and Intestinal Health of Largemouth Bass, *Micropterus salmoides*

**DOI:** 10.1155/2024/6682798

**Published:** 2024-01-18

**Authors:** Yi Wang, Jianjun Wu, Luoxin Li, Yuanfeng Yao, Chiqing Chen, Yucong Hong, Yi Chai, Wei Liu

**Affiliations:** ^1^The College of Agriculture/College of Animal Sciences, Yangtze University, Jingzhou 434020, China; ^2^Yangtze River Fisheries Research Institute, Chinese Academy of Fishery Sciences, Wuhan 430223, Hubei, China; ^3^Wuhan SunHY Biology, Wuhan 430074, Hubei, China; ^4^Wufeng Chicheng Biotech Co. Ltd., Yichang, Hubei, China; ^5^Guangdong Provincial Key Laboratory of Aquatic Larvae Feed, Guangdong Yuequn Biotechnology Co. Ltd., Jieyang, Guangdong, China

## Abstract

We investigated the effects of dietary tannic acid (TA) supplementation of a high-carbohydrate diet on growth, feed utilization, whole-body proximate composition, serum biochemical indicators, antioxidant capacity, digestive enzyme activity, and liver and intestinal health of juvenile largemouth bass, *Micropterus salmoides* (initial mean weight: 8.08 ± 0.08 g). Five diets were prepared, including a positive control (dietary carbohydrate level, 16%, LC0), a negative control (dietary carbohydrate level, 21%, HC0), and three TA-supplementation diets based on the negative control diet with TA addition at 200, 400, and 800 mg/kg, respectively. After 8 weeks of feeding, the results showed that compared with the LC0 diet, 400–800 mg/kg dietary TA significantly improved the survival rate of largemouth bass (*P* < 0.05) while significantly reducing its weight-gain rate and specific growth rate (*P* < 0.05). Compared with the HC0 diet, 400 mg/kg dietary TA significantly increased serum catalase activity (*P* < 0.05), and significantly decreased serum malondialdehyde, liver glycogen, lightness (L ^*∗*^), and yellowness (b ^*∗*^) (*P* < 0.05). Moreover, compared with the HC0 diet, 200–400 mg/kg dietary TA effectively improved the vacuolation of hepatocytes caused by the high-carbohydrate diet and reduced the occurrence of intestinal epithelial cell vacuolation and necrosis. In turn, 800 mg/kg dietary TA significantly inhibited protease activity in the pyloric caecum and intestine (*P* < 0.05). In conclusion, dietary supplementation with TA inhibited protease activity, which resulted in decreased growth performance in largemouth bass. However, it was also found that 200–400 mg/kg TA enhanced the antioxidant capacity of largemouth bass in the case of the high-carbohydrate diet, reduced liver glycogen levels, and improved liver and intestinal health. Finally, it should be noted that, when the dietary TA level exceeded 800 mg/kg, TA appeared to play a pro-oxidation role in the liver, which may cause oxidative stress in the liver.

## 1. Introduction

Currently, because of the high price of fish meal and production–process limitations, it is necessary to supplement the aquatic puffed feed with a certain level of plant protein raw materials, which also makes it easy for the carbohydrate levels in the diet to exceed the tolerance of carnivorous fish [1, 2]. As a typical carnivorous fish, largemouth bass (*Micropterus salmoides*) is an important freshwater farmed fish with great economic value in China [3]. This species has a poor carbohydrate-utilization capacity [4], with the carbohydrate content in its diet should not exceed 20% [5]. When a diet excessively high in carbohydrates is consistently provided largemouth bass with over an extended period, its growth is inhibited and it suffers from oxidative stress [4, 5]. In addition, a high-carbohydrate diet causes excessive accumulation of glycogen and lipid content in the liver of largemouth bass [6], resulting in hypertrophy, a pale color, and an increased vacuole area in the liver [7], causing liver functional damage [8]. Moreover, an excessive dietary carbohydrate intake altered the intestinal morphology of largemouth bass, increased the expression of genes associated with intestinal inflammation, and reduced the intestinal resistance to harmful microorganisms [9]. Therefore, identifying approaches to improve the adaptability of largemouth bass to carbohydrates in the feed and improve its liver and intestinal health is particularly important for the development of large-scale culture of largemouth bass.

Tannic acid (TA) is a type of natural polyphenolic plant, component that is mainly found in legumes, gallnuts, and other plants [10, 11]. Studies have shown that dietary supplementation of low level of TA can improve the growth performance and health status of livestock and poultry [12–14] and has a hypoglycemic effect by regulating the activity of carbohydrate metabolism enzymes [15]. In addition, TA has been found to improve liver health [16]. At present, there are few reports on the use of TA in the diet of aquatic animals. For example, in the cases of shrimp (*Litopenaeus vannamei*) and pearl gentian grouper (*♂ Epinephelus lanceolatus × ♀ E. fuscoguttatus*), dietary TA at 1,000 mg/kg significantly improved growth performance, antioxidant capacity, and intestinal health [17, 18]. In Japanese seabass (*Lateolabrax japonicus*), 400 mg/kg dietary TA improved the antioxidant capacity without affecting growth and reduced the cumulative mortality under hypoxic stress by activating the Nrf2 signaling pathway [19]. In grass carp (*Ctenopharyngodon idellus*), dietary 1.25% TA promoted the utilization of carbohydrates by increasing amylase activity and reducing lipid deposition [20]. Although the studies mentioned above showed that dietary TA has promoting effects in aquatic animals similar to those observed in livestock and poultry, it remains unknown whether dietary TA has the same effect in fish fed with a high-carbohydrate diet, especially in the case of carnivorous fish, such as largemouth bass.

Therefore, in the present study, an 8-week feeding trial was conducted to evaluate the effects of TA supplementation in a high-carbohydrate diet on growth performance, body composition, antioxidant capacity, digestive enzyme activity, and histological morphology of the liver and intestine in largemouth bass.

## 2. Materials and Methods

All care and handling procedures of largemouth bass performed in the present study were approved by the Animal Care and Use Committee of Yangtze University.

### 2.1. Diets

Five isonitrogenous and isolipidic experimental diets were designed. One was a low-carbohydrate diet (16% carbohydrates, termed LC0). The four high-carbohydrate diets were supplemented with 0, 200, 400, and 800 mg/kg TA, respectively (21% carbohydrates, termed HC0, HC200, HC400, and HC800, respectively). The formulation and approximate compositions of the experimental diets are shown in Table 1.

All ingredients were ground into a powder through a 60-mesh sieve. Water and oil were added to the premixed dry ingredients and were thoroughly mixed until homogenous. Subsequently, pellets with a diameter of 2 mm were extruded using a twin-screwed extruder (TSE65, Yanggong Machinery Technology Development Co., Ltd., Beijing, China), then dried with a belt dryer (DW, Sushi Drying Equipment Co., Ltd., Changzhou, China) at 70–80°C for 15 min. After drying, all diets were sealed in a plastic bag and stored in a freezer (–20°C) until use.

### 2.2. Fish and Culture Conditions

Juvenile largemouth bass were obtained from a commercial hatchery (Jingzhou, Hubei Province, China). The fish were acclimated and fed dietary LC0 for 2 weeks. At the beginning of the formal feeding experiment, all fish were fasted for 24 hr. Then, 600 healthy fish with a similar size (8.08 ± 0.08 g) were selected and randomly stocked in 15 cylindrical tanks (270 L) with circulating water, at a density of 40 fish per tank. The water used for aquaculture was groundwater. The 15 tanks were randomly divided into five groups with three replicates; the five types of experimental diets were fed to these groups, respectively. During the 8-week feeding trial, the fish were fed twice daily (08 : 00 and 16 : 30) until apparent satiation, as assessed based on visual observation. The water temperature, feed consumption, and number and weight of dead fish were recorded daily. The aquaculture water was exchanged once a day, with each exchange volume accounting for one-fifth of the total water volume. During the feeding experiment, the water temperature ranged from 25.0 to 30.0°C, the dissolved oxygen varied from 6.0 to 6.5 mg/L, the pH ranged from 6.8 to 7.3, and total ammonia was <0.01 mg/L.

### 2.3. Sampling and Analysis

#### 2.3.1. Sample Collection

At the end of the feeding experiment, the fish were fasted for 24 hr. Subsequently, three fish were randomly taken from each tank and anesthetized with 0.1 g/kg MS-222 (Sigma, USA). First, the body length and weight of the animals were measured, to determine the condition factor (CF). Second, blood was collected from the tail vein, equal volumes from each fish were mixed, and the mixed blood was left standing in a 4°C refrigerator for 2 hr; it was then centrifuged at a speed of 1,000x *g* for 15 min, and the supernatant was collected and placed in a −80°C freezer for the determination of serum biochemical indicators. Third, the viscera and liver were dissected from the three fish, weighed, and recorded for the calculation of the viscerosomatic and hepatosomatic indexes (HSIs). Fourth, samples of the tissues from the liver tip and middle intestine of each fish were collected and placed in 4% paraformaldehyde, to prepare tissue slices. Fifth, samples of the tissues from the liver tip and middle intestine were collected and mixed, for the determination of antioxidant enzyme indexes, digestive enzymes, and liver glycogen. Moreover, the remaining liver tissues were mixed and stored for the determination of proximate composition. Sixth, the muscles above the lateral line were dissected from the back of the three fish, and the muscle samples were preserved at −20°C for the determination of muscle proximate composition. Seventh, three fish were randomly collected from each tank and the liver was dissected to determine the liver color. Lastly, three fish were randomly collected from each tank and stored at −20°C for the determination of the whole-body proximate composition. Finally, the total number and body weight of the remaining fish in each tank were measured, respectively, to calculate the survival, growth performance, and feed utilization of these largemouth bass.

#### 2.3.2. Diets and Fish Body Composition Analysis

The proximate compositions of the experimental diets and fish samples (whole body, liver, and muscle) were analyzed using the standard methods of the Association of Official Analytical Chemists [21]. The diet and fish samples were dried to a constant weight at 105°C, to determine the moisture content. The Kjeldahl method using a semiautomatic digester (K9840, Hanon Advanced Technology Group Co., Ltd., China) was adopted to analyze the crude protein content. The crude lipid content was determined via the Soxhlet extraction method, whereas the ash content was determined by combustion at 550°C to a constant weight. In turn, the carbohydrate content in the diet was determined using the 3,5-dinitrosalicylic acid method. Finally, the muscle and liver glycogen levels were measured using commercial assay kits (A043-1-1, Nanjing Jiancheng Bioengineering Institute, China) in accordance with the manufacturer's instructions.

#### 2.3.3. Determination of Serum Biochemical Indexes

The liver and intestinal samples were homogenized with saline solution in a ratio of 1 : 9 (g/mL) on ice. The homogenate was centrifuged (2,500x *g*, at 4°C) for 10 min, and the supernatant was collected and stored at −80°C for analyses.

Before the determination of serum biochemical indexes, samples of frozen serum, liver, and intestinal homogenate were thawed at 4°C. Aspartate aminotransferase (AST), alanine aminotransferase (ALT), alkaline phosphatase (ALP), triglycerides (TG), total protein (TP), albumin (ALB), total cholesterol (TC), high-density lipoprotein cholesterol (HDL-C), low-density lipoprotein cholesterol (LDL-C), lipase (LIP), and *α*-amylase (*α*-Amy) were determined using a automatic biochemical analyzer (BS-460, Shenzhen Mindray Biomedical Electronics Co., Ltd., Shenzhen, China), and the reagents used were the same batch of reagents purchased from Shenzhen Mindray Biomedical Electronics Co., Ltd., Shenzhen, China.

#### 2.3.4. Determination of Antioxidant Indexes in the Serum and Liver

Antioxidant indicators, including malondialdehyde (MDA), catalase (CAT), and superoxide dismutase (SOD), were measured using commercial assay kits (Nanjing Jiancheng Bioengineering Institute, Nanjing, China) in accordance with the manufacturer's instructions. The catalog numbers were as follows: MDA, A003-1-2; CAT, A007-1-1; SOD, A001-3-2.

#### 2.3.5. Histological Analysis of the Liver and Intestine

The liver and intestine were fixed with 4% paraformaldehyde for 24 hr. The tissues were routinely embedded in paraffin and cut into blocks, followed by the cutting of the blocks into 6-*μ*m longitudinal sections and staining with hematoxylin and eosin.

Paraffin sections of the liver and intestinal tissue samples were observed using an optical microscope (CX33, Olympus Corporation, Japan), and images were acquired using an imaging system. The images were then used to measure the length of liver cells, cell width, nuclear diameter, villi height of the intestinal tissue, villi width, and muscle-layer thickness with the Image Pro Plus 9.0 software. Four photos of each tissue were randomly selected for analysis, and the measurement method was as described in Liu et al.'s [22] study.

The color of the liver tissue was measured by a colorimeter (WSC-1B, Shanghai Yidian Scientific Instrument Co., Ltd., Shanghai, China). For this, the liver sample was placed on a clean filter paper, the probe of the colorimeter was aligned with the liver sample, and the lightness (L ^*∗*^), redness (a ^*∗*^), and yellowness (b ^*∗*^) of the samples were measured.

#### 2.3.6. Digestive Enzyme Determination

The protease activity of the tissue samples was determined using commercial kits (A080-2-2, Nanjing Jiancheng Bioengineering Institute, Nanjing, China), according to their instructions. Lipase (LIP) and *α*-amylase (*α*-Amy) were determined on an automatic biochemical analyzer (BS-460, Shenzhen Mindray Biomedical Electronics Co., Ltd., Shenzhen, China), and the reagents used were the same batch of reagents purchased from Shenzhen Mindray Biomedical Electronics Co., Ltd., Shenzhen, China.

### 2.4. Calculation Formulas

The survival rate (SR), weight-gain rate (WGR), specific growth rate (SGR), feed-conversion ratio (FCR), feed intake (FI), CF, visceral somatic index (VSI), and HSI were calculated using the following formulas:

SR (%) = 100 × (final amount of fish/initial amount of fish).

WGR (%) = 100 × (final weight (g) − initial weight (g))/initial weight (g).

SGR (%/day) = 100 × (ln final weight − ln initial weight)/days of feeding.

FCR = total feed intake (g, dry matter)/(final weight − initial weight).

FI (%) = 100 × total amount of feed consumed/((initial weight + final weight)/2 × days of feeding).

CF = 100 × (body weight (g)/total length^3^ (cm)).

VSI (%) = 100 × (viscera weight (g)/body weight (g)).

HSI (%) = 100 × (hepatic weight (g)/body weight (g)).

### 2.5. Data Processing and Statistical Analysis

All experimental data were collated by Excel 2019 and statistically analyzed using the SPSS 22.0 software (IBM Corporation, New York, USA). All experimental data were analyzed by one-way analysis of variance. Turkey's test was used for further multiple comparisons, with a significance level of 0.05. The results are presented as the mean ± standard error (mean ± SE) of three replicates.

## 3. Results

### 3.1. Growth Performance and Feed Utilization

Compared with the LC0 group, the increase in the dietary TA level triggered a significant increase in the SR of the fish in the HC400 and HC800 groups (*P* < 0.05); in contrast, FW, WGR, and SGR were significantly decreased in these groups (*P* < 0.05). Compared with the HC0 group, the FW in the HC400 and HC800 groups was significantly reduced (*P* < 0.05) (Table 2).

### 3.2. Whole-Body, Muscle, and Liver Proximate Composition

The whole-body, muscle, and liver proximate composition of the fish are presented in Table 3. Compared with the LC0 group, dietary supplementation with 400 mg/kg TA significantly reduced the ash content of whole fish (*P* < 0.05). In turn, compared with the HC0 group, dietary TA supplementation had no significant effects on the contents of moisture, crude protein, crude lipid, and ash of whole fish (*P* > 0.05), whereas 400–800 mg/kg dietary TA significantly reduced the liver glycogen content (*P* < 0.05), and 800 mg/kg dietary TA significantly increased the muscle crude lipid content (*P* < 0.05).

### 3.3. Serum Biochemical Indexes

With the exception of the serum TG level, the dietary TA had no significant effect (*P* > 0.05) on ALT, AST, ALP, TP, LDL-C, HDL-C, TC, LIP, *α*-AMY, and ALB levels in largemouth bass (Table 4). After supplementing TA with high-carbohydrate diet, the serum TG level decreased with the increase of TA level in diet. The serum TG level in the HC800 group was significantly lower than that observed in the HC0 group (*P* < 0.05).

### 3.4. Antioxidative Abilities of the Serum and Liver

The effects of TA supplementation on the antioxidative abilities of serum and liver of the largemouth bass are shown in Table 5. The results showed that dietary TA had no significant effect on the activity of superoxide dismutase (SOD) (*P* > 0.05). Compared with the HC0 group, 400 mg/kg dietary TA significantly increased serum catalase (CAT) activity (*P* < 0.05) and decreased serum malondialdehyde (MDA) content (*P* < 0.05) in the serum, whereas 200 mg/kg dietary TA significantly increased CAT activity in the liver (*P* < 0.05).

### 3.5. Liver, Pyloric Caecum, and Intestinal Digestive Enzyme Activity

Compared with the HC0 group, dietary TA supplementation had no significant effect (*P* > 0.05) on the activities of *α*-amylase and lipase in the liver, pyloric caeca, and intestine (Table 6). However, the increase in the dietary TA level triggered an increase in the liver protease activity, whereas the pyloric cecum and intestinal protease activities were decreased. Moreover, 200–400 mg/kg dietary TA significantly increased the liver protease activity (*P* < 0.05) and 800 mg/kg dietary TA significantly decreased the pyloric caecum and intestinal protease activity of largemouth bass (*P* < 0.05).

### 3.6. Liver Histology, Color, and Hepatocyte Morphology

**Figure 1:**
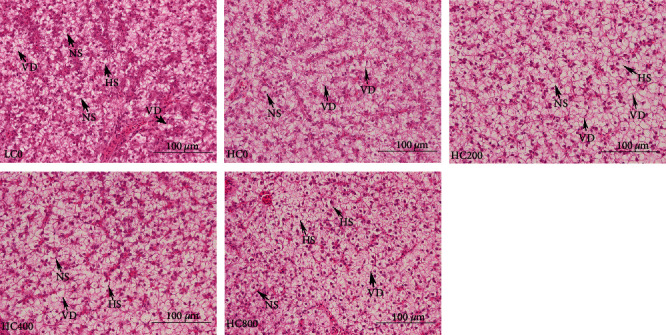
Classical liver sections of largemouth bass fed diets supplementation with tannic acid levels (H&E staining × 400, Bar = 100 *μ*m). VD, vacuolar degeneration; HS, hepatocyte swelling; and NS, nuclear shift.

**Figure 2:**
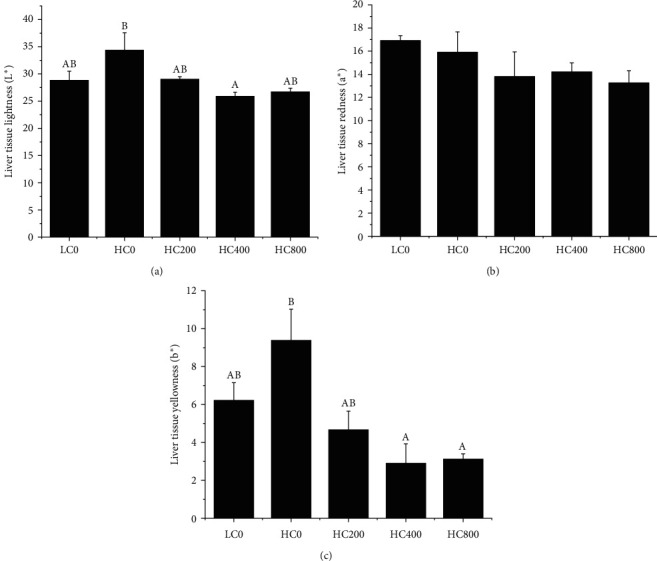
Liver tissue color from largemouth bass fed with different levels of tannic acid. (a) Liver tissue lightness (L ^*∗*^), 0–100 represents from black to white; (b) liver tissue redness (a ^*∗*^); and (c) liver tissue yellowness (b ^*∗*^). The different letters above the bar chart show the significant differences between treatments. Bar charts marked with different letters indicated significant differences (*P* < 0.05). Values are expressed as mean ± SE.

The liver histology of largemouth bass is reported in Figure 1. The tissue structure of the liver in each group was relatively complete, and the cell membrane and nucleus of liver cells were clearly visible. However, hepatocyte swelling and nuclear displacement to the edge were observed in all hepatocytes in each group, with varying degrees of vacuolation. Compared with the HC0 group, 200–400 mg/kg TA diet reduced the occurrence of the hepatocyte vacuolation. Moreover, the results of liver color determination showed that 400 mg/kg dietary TA significantly decreased the brightness (L ^*∗*^) and yellowness (b ^*∗*^) of the liver (*P* < 0.05) (Figure 2). Finally, as reported in Table 7, dietary TA had no significant effect on the hepatocyte morphometric parameters (*P* > 0.05).

### 3.7. Histology and Morphology of the Intestine

**Figure 3:**
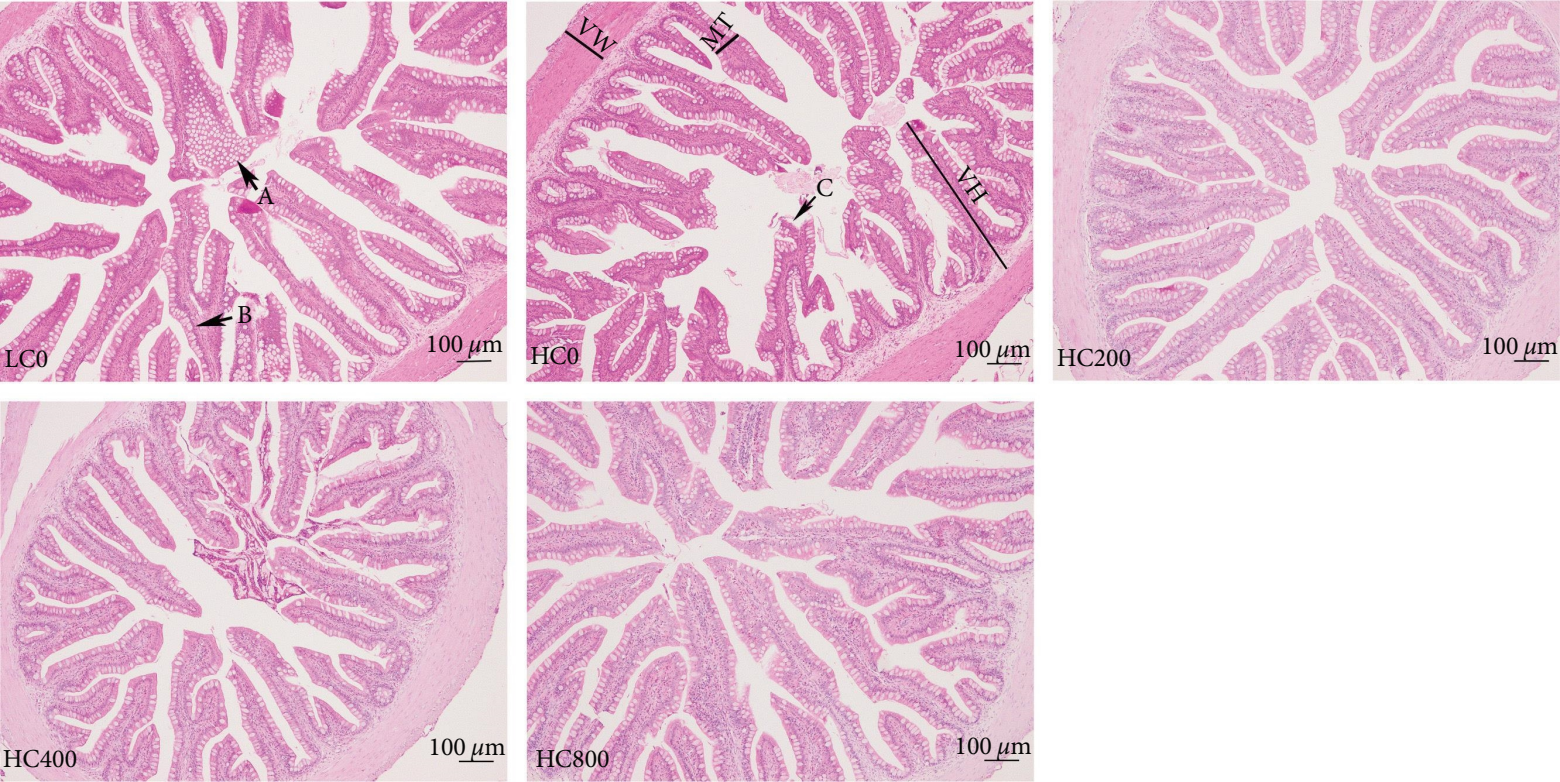
Classical intestinal sections of largemouth bass fed diets supplementation with tannic acid levels (H&E staining × 100, Bar = 100 *μ*m). A, vacuolization of intestinal epithelial cells; B, goblet cells proliferate; and C, intestinal epithelial cell necrosis. VH, villus height; VW, villus width; and MT, muscles thickness.

As depicted in Figure 3, vacuolization of intestinal epithelial cells and proliferation of epithelial cells and goblet cells were common among fish in the LC0 and HC0 groups, with some intestinal epithelial cell necrosis even being observed in the HC0 group. However, these symptoms were significantly reduced or abrogated after the largemouth bass were fed with diets containing TA.

**Figure 4:**
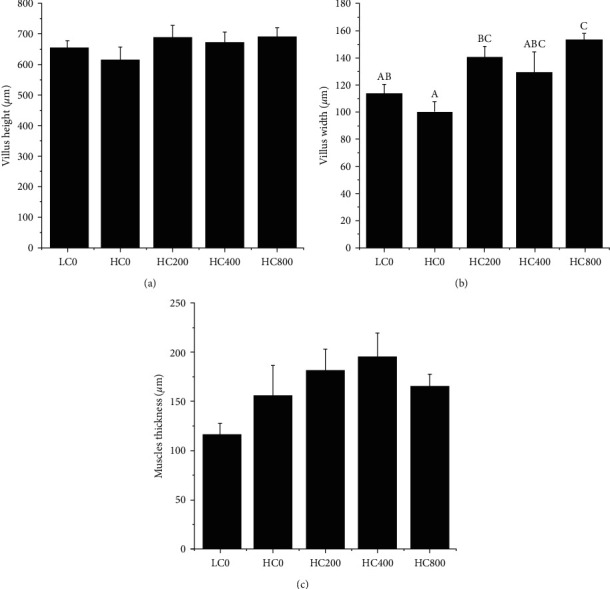
Intestinal morphology from largemouth bass fed with different levels of tannic acid: (a) villus height, (b) villus width, and (c) muscles thickness. The different letters above the bar chart show the significant differences between treatments. Bar charts marked with different letters indicated significant differences (*P* < 0.05). Values are expressed as mean ± SE.

Supplementation of the diets with TA had no significant effect (*P* > 0.05) on the villus height and muscle layer thickness of the largemouth bass (Figure 4). However, diet supplementation with TA triggered a trend toward an increase in the villus height, villus width, and muscle-layer thickness of the intestinal tract of largemouth bass, especially regarding the villus width of the fish in the HC200 and HC800 groups, which was significantly higher than that of the HC0 group (*P* < 0.05).

## 4. Discussion

### 4.1. Effects of TA Levels on the Growth Performance, Feed Utilization, and Digestive Enzyme Activities of Largemouth Bass

In this study, dietary TA supplementation improved the SR of largemouth bass, which was consistent with the results of similar research performed on shrimp [17]. This may be attributed to the bacteriostatic effect of dietary TA, which can effectively reduce the toxic effect of harmful microorganisms and improve the immune performance of fish, thus improving the SR [23, 24]. TA has the effect of improving growth performance and is currently commonly used as a growth promoter in the livestock and poultry-breeding industries [25, 26]. In aquatic animals, studies of shrimp and pearl gentian grouper also reported that the WGR and SGR were significantly increased at a dietary TA level of 1,000 mg/kg [17, 18]. In addition, in the beluga sturgeon (*Huso huso*), dietary TA significantly upregulated the expression of the growth hormone and insulin-like growth factor (IGF-Ⅰ) genes, which may help explain the promoting effect of TA on fish growth [27]. However, in the present study, 400–800 mg/kg dietary TA significantly reduced the WGR and SGR of the fish compared with the LC0 group, and the growth of largemouth bass was inhibited. This growth retardation may have been caused by the high-carbohydrate diet [28], but dietary TA seemed to exacerbate this negative effect of the high-carbohydrate diet on the growth of largemouth bass. Some studies have reported that excessive levels of dietary TA may reduce the palatability of the diet, resulting in reduced dietary intake and thus the growth retardation in fish [29–31]. In contrast, in the present study, there was no significant difference in FI and FCR among the groups of largemouth bass, and TA had no significant effect on the feed utilization of largemouth bass. Therefore, we can conclude that the decrease of growth performance of largemouth bass was not caused by the change in dietary palatability caused by TA, and we speculated that it might be related to the inhibition of TA on digestive enzyme activity.

The determination of digestive enzyme activity is usually used to evaluate the capacity of fish to digest and absorb nutrients [32]. In this study, compared with the HC0 group, the protease activity of the pyloric caecum and intestine of largemouth bass were decreased after dietary TA supplementation. This occurred because TA can combine with exogenous proteins to form insoluble complexes, inhibit protease activity, and decrease protein digestibility [33]. We believe that this may be the main reason for the decreased growth performance of largemouth bass observed in this study. Unexpectedly, protease activity in the liver, in this study, was significantly increased when the dietary TA levels were 200–400 mg/kg, which may represent a compensatory adjustment. Because TA inhibited the protease activity of the pyloric caecum and intestine, the body would require that the pancreas and the liver secrete more proteases to compensate for the adverse effects of TA on the digestion and absorption of proteins [34, 35]. In addition, the results obtained in grass carp showed that, although 1.25% dietary TA impaired protein metabolism, it was also found that dietary TA increased amylase activity, thereby promoting the digestion and absorption of carbohydrates in fish [20]. Similar results were obtained in obscure puffer [34]. This may be attributed to the fact that TA stimulates sympathetic pathways and adrenaline, thus promoting the expression of amylase-related genes [36]. In this study, it was found that 200–400 mg/kg TA increased amylase activity in the pyloric caecum of largemouth bass compared with the HC0 group, although this difference was not statistically significant. We suggest that the increase in amylase activity is a countermeasure adopted by fish against the inhibition of protein metabolism caused by TA.

### 4.2. Effects of Dietary TA Levels on Body Lipid Deposition and Blood Lipid Indicators of Largemouth Bass

Previous studies have demonstrated that dietary supplementation with TA can significantly reduce the crude lipid content of fish, resulting in leaner fish [30, 37]. This is because dietary TA may form a complex with enzymes that are involved in lipid metabolism, thus inhibiting lipid synthesis or lipid mobilization, impairing the utilization of lipids in the diet, and leading to a decrease in body lipid deposition [38]. However, in the present study, it was observed that 800 mg/kg dietary TA significantly increased the muscle crude lipid content of largemouth bass compared with the HC0 diet. We believe that this is related to the decrease of serum TG levels. As an important blood lipid indicator, TG levels can reflect the body's ability to absorb and metabolize lipids [39]. Previous studies have reported that TA can stimulate the transformation of TG in the blood or inhibit the synthesis of TG in the liver [40]. We hypothesize that TA promotes the conversion of TG in the blood of largemouth bass and is deposited in the muscle as a lipid, thus leading to an increase in the crude lipid content in the muscle. In fish, the risk of developing a fatty liver increases when the serum TG levels are excessive [41]. The significant reduction of TG also suggests that dietary TA can improve the high lipid levels caused by the high-carbohydrate diet and promote the blood lipid health of largemouth bass.

### 4.3. Effects of Dietary TA Levels on Liver Health and Antioxidant Capacity of Largemouth Bass

TA, as a polyphenol compound, has a strong antioxidant activity, which can effectively remove a variety of free radicals and reactive oxygen species to reduce oxidative stress by activating antioxidant enzymes [27]. Many livestock studies have confirmed the antioxidant potential of TA [42, 43]; nevertheless, few studies have addressed the effect of TA on the antioxidant capacity of fish. In Japanese seabass, 400 mg/kg dietary TA reportedly increased CAT and SOD activity in the serum and liver and decreased serum MDA levels [19]. A study of pearl gentian grouper also confirmed that TA can promote the mRNA expression of genes related to antioxidant enzymes (CAT, Mn-SOD, etc.), improve the activity of antioxidant enzymes, and, thus, enhance the antioxidant capacity of fish [23]. Similarly, in this study, it was also found that dietary TA at 400 mg/kg significantly increased the serum CAT activity and significantly decreased the serum MDA levels, suggesting that dietary TA can help reduce the oxidative stress caused by the high-carbohydrate diet. However, it is worth noting that, compared with the HC0 group, the CAT activity in the liver increased sharply at 200 mg/kg dietary TA, but plummeted at 800 mg/kg dietary TA; moreover, the liver MDA levels in the groups that received TA supplementation were increased to some extent, although this difference was not statistically significant. These results were unexpected, as TA, which has strong antioxidant properties, seemed to affect negatively the antioxidant capacity of the liver of largemouth bass. Previous studies have shown that, because of the astringent effect of TA, this compound can combine with foreign proteins and lipids to form TA–protein (–lipid) complexes, which are easily oxidized by oxygen ions or hydrogen peroxide, resulting in the production of free radicals, thus explaining the pro-oxidation effect of TA in this context [44]. In obscure puffer (*Takifugu fasciatus*), it was found that the liver MDA level was significantly increased and that the liver HSP70 expression was significantly decreased at a dietary TA level of 1.25%. This indicates that the liver of obscure puffer undergoes oxidative stress after dietary TA supplementation [34]. This is consistent with the findings in grass carp [20]. In the present study, we believe that dietary TA had a pro-oxidation effect on the liver of largemouth bass. Regarding the pro-oxidation effect of TA in the liver, although the body increased the liver CAT activity correspondingly as the dietary TA level increased, it was still not sufficient to offset the oxidative stress caused by TA in the liver. This may explain the sharp decline in liver CAT activity observed when dietary TA was administered at 800 mg/kg.

Liver color is usually an important indicator that is used to judge fish health, and the appearance of a healthy liver is mostly bright red [45]. In carp (*Cyprinus carpio* L.), the liver tissue was bright red after intraperitoneal injection of 10 mg/kg of body weight of TA. In addition, compared with the severe liver necrosis observed after CuSO_4_ treatment alone, the liver necrosis of carp was improved after combined treatment with TA together with CuSO_4_, and the liver color was less pale [46]. In the current study, 400 mg/kg TA significantly reduced the lightness (L ^*∗*^) and yellowness (b ^*∗*^) of the liver, indicating the presence of a relatively redder liver color, which is generally considered to be a sign of liver health. The liver is the main organ for the carbohydrate metabolism of fish, and a high-carbohydrate diet facilitates the abnormal deposition of glycogen and lipids in the liver, leading to the vacuolization of hepatocytes, and damaging liver health [47]. In grass carp, it was found that liver cells became more compact, and hepatocyte vacuolation was decreased as the dietary TA level increased [48]. The results of liver histology obtained in the present study also showed that 200–400 mg/kg TA effectively reduced the vacuolation of largemouth bass hepatocytes compared with the HC0 group. It is generally believed that vacuoles contain lipids and glycogen, which are related to the normal metabolic function of the liver. We propose that the improvement of hepatocyte vacuolation in largemouth bass in this study is related to the decrease in the glycogen levels in the liver. In this study, TA significantly reduced liver glycogen levels and reduced liver glycogen deposition, which had a positive effect on the liver health of largemouth bass fed with a high-carbohydrate diet [49].

### 4.4. Effects of Dietary TA Levels on the Intestinal Histology and Morphology of Largemouth Bass

The intestine is the main organ for digestion and absorption in fish, thus playing a crucial role in nutrient absorption and metabolism [50]. In previous reports, TA, as an antinutritional factor, was suggested to potentially impair intestinal health [51]. With the deepening of research, in Japanese seabass, it was found that dietary TA not only does not affect the integrity of the intestinal tract but also has an anti-inflammatory effect, which can effectively reduce the concentration of endotoxins and reduce intestinal inflammation, thus promoting intestinal health [52]. In this study, adverse conditions, such as vacuolization of epithelial cells and goblet cell hyperplasia, and even necrosis of epithelial cells, were observed in the intestine of largemouth bass that were fed a high-carbohydrate diet, whereas TA supplementation reduced the occurrence of the intestinal adverse reactions caused by the high-carbohydrate diet. In addition, the intestinal protection afforded by dietary TA is also reflected in the improvement of intestinal morphology [53, 54]. Research has shown that dietary TA can effectively inhibit the protein decomposition reaction of harmful microorganisms in the intestine, thereby reducing the toxic effect of ammonia on intestinal cells and improving the morphology of the intestinal tissue [55]. In the pearl gentian grouper, dietary TA was found to significantly increase the height of intestinal villi, and the intestinal villi were more tightly arranged, indicating improved intestinal health [23]. In this study, the intestinal morphological results also showed that 200 mg/kg TA significantly increased the width of intestinal villi, which means that the nutrient absorption area was increased [56], and has a positive effect on the intestinal health of largemouth bass fed with a high-carbohydrate diet.

## 5. Conclusion

The results of this study suggest that supplementation of a high-carbohydrate diet with dietary TA can inhibit protease activity in the pyloric caecum and intestine of largemouth bass and negatively affect growth performance. However, it was also found that 200–400 mg/kg dietary TA increased CAT activity, reduced serum MDA levels, decreased liver glycogen deposition, and improved liver and intestinal tissue structure. Moreover, 800 mg/kg TA can significantly reduce the serum TG level, but it should be noted that a dietary TA level that exceeds 800 mg/kg appears to play a pro-oxidation role in the liver, which may cause oxidative stress in the liver of largemouth bass.

## Figures and Tables

**Table 1:** The formulation and proximate compositions of the experimental diets (g/kg diet).

Items	Diets				
	LC0	HC0	HC200	HC400	HC800
Fish meal	488.50	488.50	488.50	488.50	488.50
Chicken meal	90.00	90.00	90.00	90.00	90.00
Plasma protein powder	50.00	50.00	50.00	50.00	50.00
Wheat gluten	30.00	30.00	30.00	30.00	30.00
Soybean meal	60.00	60.00	60.00	60.00	60.00
Peanut meal	25.00	20.00	20.00	20.00	20.00
Rice bran	20.00	10.00	9.80	9.60	9.20
Tannic acid premix^a^	0.00	0.00	0.20	0.40	0.80
Wheat flour	40.00	70.00	70.00	70.00	70.00
Cassava starch	40.00	70.00	70.00	70.00	70.00
Yeast	10.00	10.00	10.00	10.00	10.00
Bentonite	50.00	5.00	5.00	5.00	5.00
Ca(H_2_PO_4_)_2_	15.00	15.00	15.00	15.00	15.00
Fish oil	30.00	30.00	30.00	30.00	30.00
Soybean oil	30.00	30.00	30.00	30.00	30.00
Vitamin premix^b^	10.00	10.00	10.00	10.00	10.00
Mineral premix^c^	10.00	10.00	10.00	10.00	10.00
Choline chloride	1.50	1.50	1.50	1.50	1.50
Total	1,000.00	1,000.00	1,000.00	1,000.00	1,000.00
Proximate analysis (dry matter)					
Crude protein	531.00	531.60	530.80	531.30	532.50
Crude lipid	100.10	100.80	100.50	100.60	100.30
Ash	204.30	153.70	151.30	156.40	153.40
Carbohydrate	164.60	213.90	217.40	211.70	213.80

^*a*^The premix contained 80% hydrolyzed Chinese Gallnut (Galla Chinensis) tannic acid, which was obtained from Wufeng Chicheng Biotech Co., Ltd., China. ^b^Vitamin premix (g/kg diet): VA 0.0048, VD_3_ 0.2, VE 0.16, VK_3_ 0.01472, VC 0.08, VB_1_ 0.0178, VB_2_ 0.048, VB_6_ 0.0295, VB_12_ 0.00024, inositol 0.032, pantothenate 0.0736, niacinamide 0.0792, folic acid 0.0064, and biotin 0.00064. ^c^Mineral premix (g/kg diet): KH_2_PO_4_ 1.35, NaCl 0.05, MgSO_4_ · 7H_2_O 0.75, KI 0.0015, CuSO_4_ · 5H_2_O 0.015, ZnSO_4_ · 7H_2_O 0.175, FeSO_4_ · 7H_2_O 1.25, MnSO_4_ · 4H_2_O 0.08, Na_2_SeO_3_ 0.001, Ca(H_2_PO_4_)_2_ 1.8, and NaH_2_PO_4_ · 2H_2_O 0.65.

**Table 2:** Effects of dietary tannic acid levels on growth performance and feed utilization of largemouth bass.

Items	Diets				
	LC0	HC0	HC200	HC400	HC800
IW (g)	8.08 ± 0.08	8.13 ± 0.26	8.09 ± 0.19	8.13 ± 0.18	8.02 ± 0.00
FW (g)	32.73 ± 0.91^c^	30.97 ± 0.79^bc^	27.10 ± 0.84^ab^	26.97 ± 0.24^a^	26.84 ± 1.22^a^
SR (%)	91.67 ± 0.83^a^	96.67 ± 2.20^ab^	97.50 ± 1.44^ab^	100.00 ± 0.00^b^	99.17 ± 0.83^b^
WGR (%)	304.86 ± 9.43^b^	282.57 ± 21.97^ab^	235.74 ± 15.51^a^	232.17 ± 6.49^a^	234.68 ± 15.13^a^
SGR (%/day)	2.50 ± 0.04^b^	2.39 ± 0.10^ab^	2.16 ± 0.08^ab^	2.14 ± 0.04^a^	2.15 ± 0.04^a^
FI (%/day)	1.82 ± 0.06	1.73 ± 0.07	1.64 ± 0.05	1.80 ± 0.08	1.73 ± 0.09
FCR	0.91 ± 0.04	0.85 ± 0.03	0.87 ± 0.05	0.94 ± 0.04	0.91 ± 0.07
CF (g/cm^3^)	2.15 ± 0.05	2.22 ± 0.12	2.10 ± 0.05	2.21 ± 0.03	2.25 ± 0.10
VSI (%)	6.23 ± 0.31	6.40 ± 0.15	6.44 ± 0.33	6.60 ± 0.25	6.48 ± 0.19
HSI (%)	0.98 ± 0.08	1.06 ± 0.06	1.06 ± 0.22	0.96 ± 0.17	0.99 ± 0.02

IW, initial weight; FW, final weight; SR, survival rate; WGR, weight gain rate; SGR, specific growth rate; FI, feed intake; FCR, feed conversion ratio; CF, condition factor; VSI, visceral–somatic index; and HSI, hepatosomatic index. All data were shown as mean ± SE of three replicates. Values with different letters in the same row were significantly different (*P* < 0.05).

**Table 3:** Effects of dietary tannic acid levels on the whole-body, liver, and muscle proximate composition of largemouth bass (%).

Items	Diets				
	LC0	HC0	HC200	HC400	HC800
Whole body					
Moisture	68.35 ± 1.24	71.49 ± 0.40	70.99 ± 0.59	71.10 ± 0.44	71.46 ± 0.34
Crude protein	18.00 ± 0.71	17.62 ± 0.23	17.74 ± 0.59	17.72 ± 0.18	17.93 ± 0.59
Crude lipid	7.60 ± 0.45	6.29 ± 0.36	6.61 ± 0.36	6.37 ± 0.51	6.09 ± 0.11
Ash	4.34 ± 0.07^b^	4.04 ± 0.11^ab^	3.91 ± 0.03^ab^	3.69 ± 0.16^a^	4.20 ± 0.02^b^
Liver					
Moisture	79.11 ± 2.35	76.29 ± 1.12	80.09 ± 6.19	78.19 ± 2.76	75.76 ± 1.00
Crude lipid	5.47 ± 0.65^a^	2.90 ± 0.24^b^	2.28 ± 0.30^b^	3.18 ± 0.67^b^	3.85 ± 0.64^ab^
Glycogen	6.12 ± 0.23^b^	8.07 ± 0.40^c^	6.86 ± 0.37^bc^	4.60 ± 0.17^a^	4.65 ± 0.14^a^
Muscle					
Moisture	77.23 ± 0.08^ab^	77.68 ± 0.76^ab^	78.45 ± 0.35^b^	76.56 ± 0.68^a^	76.52 ± 0.50^a^
Crude lipid	0.85 ± 0.31^ab^	0.32 ± 0.02^a^	0.62 ± 0.10^ab^	0.96 ± 0.35^ab^	1.24 ± 0.24^b^
Glycogen (mg/g)	0.14 ± 0.01^a^	0.22 ± 0.01^b^	0.22 ± 0.01^b^	0.21 ± 0.01^b^	0.19 ± 0.01^b^

All data were shown as mean ± SE of three replicates. Values with different letters in the same row were significantly different (*P* < 0.05).

**Table 4:** Effect of dietary tannic acid levels on serum biochemical parameters of largemouth bass.

Items	Diets				
	LC0	HC0	HC200	HC400	HC800
ALT (U/L)	7.20 ± 1.11	8.40 ± 0.23	6.67 ± 0.29	6.53 ± 0.13	8.00 ± 0.20
AST (U/L)	57.33 ± 6.53	64.07 ± 0.29	74.53 ± 6.01	58.67 ± 2.95	62.73 ± 5.31
ALP (U/L)	78.13 ± 3.35	84.33 ± 7.36	76.40 ± 3.14	78.27 ± 4.87	75.33 ± 1.47
TP (g/L)	31.93 ± 1.44	31.87 ± 0.87	32.47 ± 1.78	30.07 ± 0.81	31.60 ± 1.33
TG (mmol/L)	2.96 ± 0.33^ab^	3.36 ± 0.21^b^	2.39 ± 0.36^ab^	2.23 ± 0.21^ab^	2.03 ± 0.26^a^
LDL-C (mmol/L)	2.27 ± 0.10	2.30 ± 0.20	2.01 ± 0.24	2.03 ± 0.07	2.07 ± 0.06
HDL-C (mmol/L)	4.45 ± 0.23	4.11 ± 0.07	4.60 ± 0.20	4.41 ± 0.11	4.39 ± 0.16
TC (mmol/L)	8.97 ± 0.45	8.67 ± 0.37	8.44 ± 0.56	8.26 ± 0.30	8.64 ± 0.21
LIP (U/L)	4.80 ± 0.31	4.67 ± 0.24	4.67 ± 0.24	4.07 ± 0.35	4.53 ± 0.33
*α*-AMY (U/L)	293.67 ± 30.87	275.53 ± 30.53	287.53 ± 23.82	282.87 ± 11.58	256.93 ± 18.03
ALB (g/L)	7.00 ± 0.53	7.67 ± 0.37	7.67 ± 0.81	6.87 ± 0.18	6.80 ± 0.35

ALT, alanine aminotransferase; AST, aspartate aminotransferase; ALP, alkaline phosphatase; TP, total protein; TG, triglyceride; LDL-C, low-density lipoprotein cholesterol; HDL-C, high-density lipoprotein cholesterol; TC, total cholesterol; LIP, lipase; *α*-AMY, *α*-amylase; and ALB, albumin. All data were shown as mean ± SE of three replicates. Values with different letters in the same row were significantly different (*P* < 0.05).

**Table 5:** Effect of dietary tannic acid levels on antioxidant indexes in serum and liver of largemouth bass.

Items	Diets				
	LC0	HC0	HC200	HC400	HC800
Serum					
SOD (U/mL)	80.13 ± 7.12	84.00 ± 1.69	75.38 ± 3.16	79.56 ± 0.78	74.09 ± 4.09
CAT (U/mL)	166.07 ± 5.66^ab^	137.52 ± 7.48^a^	239.24 ± 11.28^c^	184.88 ± 7.02^b^	140.77 ± 8.73^a^
MDA (nmol/mL)	5.09 ± 0.44^ab^	6.14 ± 0.75^b^	4.58 ± 0.05^ab^	4.20 ± 0.28^a^	4.37 ± 0.15^ab^
Liver					
SOD (U/mg protein)	15.14 ± 1.04	14.34 ± 1.01	12.94 ± 0.22	14.75 ± 1.62	14.31 ± 0.61
CAT (U/mg protein)	6.26 ± 0.14^ab^	5.93 ± 1.43^ab^	17.12 ± 1.03^c^	7.10 ± 1.22^b^	2.61 ± 0.14^a^
MDA (nmol/mg protein)	1.18 ± 0.14^a^	1.34 ± 0.20^ab^	2.15 ± 0.35^ab^	2.46 ± 0.17^b^	1.85 ± 0.32^ab^

SOD, superoxide dismutase; CAT, catalase; and MDA, malondialdehyde. All data were shown as mean ± SE of three replicates. Values with different letters in the same row were significantly different (*P* < 0.05).

**Table 6:** Effect of dietary tannic acid levels on digestive enzyme activity of liver, pyloric caecum, and intestine of largemouth bass (U/g protein).

Items	Diets				
	LC0	HC0	HC200	HC400	HC800
Liver					
* α*-Amylase	12.88 ± 1.62	6.38 ± 1.25	8.74 ± 2.37	9.17 ± 0.53	6.46 ± 1.43
Lipase	0.79 ± 0.00	0.69 ± 0.06	0.77 ± 0.09	0.74 ± 0.07	0.76 ± 0.02
Protease	0.33 ± 0.02^ab^	0.24 ± 0.04^a^	0.38 ± 0.05^b^	0.44 ± 0.01^b^	0.32 ± 0.01^ab^
Pyloric caecum					
* α*-Amylase	18.80 ± 2.04^ab^	14.02 ± 1.48^ab^	19.62 ± 0.26^ab^	21.60 ± 2.00^b^	13.40 ± 2.05^a^
Lipase	0.48 ± 0.14	0.53 ± 0.18	0.65 ± 0.20	0.48 ± 0.17	0.72 ± 0.16
Protease	3.82 ± 0.72^ab^	6.02 ± 0.59^b^	3.85 ± 0.62^ab^	3.58 ± 0.69^ab^	1.81 ± 0.19^a^
Intestines					
* α*-Amylase	12.10 ± 1.54	12.75 ± 0.94	11.04 ± 1.34	16.41 ± 0.19	12.15 ± 1.39
Lipase	0.52 ± 0.02	0.42 ± 0.19	0.81 ± 0.11	0.90 ± 0.08	0.91 ± 0.06
Protease	1.04 ± 0.20^a^	4.00 ± 0.46^b^	1.97 ± 0.66^a^	1.65 ± 0.39^a^	1.01 ± 0.07^a^

All data were shown as mean ± SE of three replicates. Values with different letters in the same row were significantly different (*P* < 0.05).

**Table 7:** Effect of dietary tannic acid levels on hepatocyte morphology of largemouth bass (*μ*m).

Items	Diets				
	LC0	HC0	HC200	HC400	HC800
Nuclear diameter	6.85 ± 0.19	6.72 ± 0.18	7.18 ± 0.13	6.93 ± 0.13	7.06 ± 0.23
Hepatocyte length	20.66 ± 0.93	19.99 ± 0.70	22.51 ± 1.13	22.25 ± 1.49	21.94 ± 0.73
Hepatocyte width	14.78 ± 0.60	14.68 ± 0.49	16.33 ± 0.82	15.22 ± 1.04	15.11 ± 0.89

All data were shown as mean ± SE of three replicates. Values with different letters in the same row were significantly different (*P* < 0.05).

## Data Availability

The data that support the findings of the present study are available from the corresponding author upon reasonable request.
